# Congruence between *International Classification of Disease, Tenth Revision (ICD-10)* code and written documentation for outpatient encounters with antibiotic prescriptions

**DOI:** 10.1017/ash.2023.270

**Published:** 2023-09-29

**Authors:** Charles Oertli, Milner Owens Staub, Sophie Katz

## Abstract

**Background:** Antimicrobial stewardship programs (ASPs) often rely on *International Classification of Diseases, Tenth Revision* (ICD-10) codes to assess antibiotic appropriateness for provider feedback. Concordance between encounter ICD-10 codes and documented indication for antibiotics based on manual chart review varies greatly (74%–95%) in the inpatient setting. Data on concordance between documented indication and ICD-10 code in the outpatient setting are scarce. **Methods:** We conducted a retrospective cohort study of 650 randomly selected outpatient encounters with antibiotic prescriptions from walk-in and retail clinics between July 15 and September 15, 2021, at Vanderbilt University Medical Center. We performed chart review to compare documented antibiotic indication to the 3 most frequent encounter-associated ICD-10 codes. Also, 12 encounters were excluded due to insufficient available written documentation. The 95% CI for proportion of encounters with concordant antibiotic indications was calculated using Stata version 15.1 software. **Results:** Of the 638 antibiotic prescriptions with written documentation available for chart review, 204 (32%) were for amoxicillin, 102 (16%) were for amoxicillin-clavulanate, 61 (10%) were for cefdinir, and 56 (9%) were for azithromycin. Overall, 540 (84.6%; 95% CI, 81.6%–87.4%) of 638 encounters had concordant antibiotic indication based on documentation in the note and associated ICD-10 for the encounter. Of the 540 encounters with concordant ICD-10 and documented indications, 348 (64%), 130 (24%), and 35 (6%) were listed as the first, second, and third ICD-10 codes, respectively. An additional 27 (5%) had a concordant ICD-10 code listed beyond the third position. In total, 125 (19.6%) of 638 encounters did not have the intended antibiotic indication as documented in the note in the 3 most frequent encounter-associated ICD-10 codes (whether a lower position or incongruent ICD-10 code with documentation). Of those 125 encounters, 42 (34%) had a documented diagnosis of strep pharyngitis, 16 (13%) had a documented diagnosis of skin or soft-tissue infection, 11 (9%) had a documented diagnosis of urinary tract infection, and 11 (9%) had a documented diagnosis of acute otitis media. **Conclusions:** Our data suggest that outpatient antimicrobial prescriptions correlate relatively well with encounter ICD-10 codes. However, most ASP prescribing goals aim to reduce inappropriate prescribing to 10% or fewer of prescriptions based on indication. Therefore, providers may not trust individual prescribing feedback that is based on data that is only correct 85% of the time. For ASPs to accurately assess prescribing and provide trusted, meaningful recommendations and specific feedback to individual prescribers, more reliable and valid data are needed. We intend to evaluate whether requiring outpatient antibiotic indications on prescriptions increases data reliability and validity.

**Disclosures:** None

